# Molecular basis of the remarkable species selectivity of an insecticidal sodium channel toxin from the African spider *Augacephalus ezendami*

**DOI:** 10.1038/srep29538

**Published:** 2016-07-07

**Authors:** Volker Herzig, Maria Ikonomopoulou, Jennifer J. Smith, Sławomir Dziemborowicz, John Gilchrist, Lucia Kuhn-Nentwig, Fernanda Oliveira Rezende, Luciano Andrade Moreira, Graham M. Nicholson, Frank Bosmans, Glenn F. King

**Affiliations:** 1 Institute for Molecular Bioscience, The University of Queensland, St. Lucia, QLD 4072, Australia; 2 School of Medical & Molecular Biosciences, University of Technology, Sydney, NSW 2007, Australia; 3 Department of Physiology & Solomon H. Snyder Department of Neuroscience, Johns Hopkins University, School of Medicine, Baltimore, MD 21205, USA; 4 Institute of Ecology & Evolution, University of Bern, CH 3012 Bern, Switzerland; 5 FIOCRUZ/Centro de Pesquisas René Rachou, Belo Horizonte, CEP 30190-002, MG, Brazil

## Abstract

The inexorable decline in the armament of registered chemical insecticides has stimulated research into environmentally-friendly alternatives. Insecticidal spider-venom peptides are promising candidates for bioinsecticide development but it is challenging to find peptides that are specific for targeted pests. In the present study, we isolated an insecticidal peptide (Ae1a) from venom of the African spider *Augacephalus ezendami* (family Theraphosidae). Injection of Ae1a into sheep blowflies (*Lucilia cuprina*) induced rapid but reversible paralysis. In striking contrast, Ae1a was lethal to closely related fruit flies (*Drosophila melanogaster*) but induced no adverse effects in the recalcitrant lepidopteran pest *Helicoverpa armigera*. Electrophysiological experiments revealed that Ae1a potently inhibits the voltage-gated sodium channel BgNa_V_1 from the German cockroach *Blattella germanica* by shifting the threshold for channel activation to more depolarized potentials. In contrast, Ae1a failed to significantly affect sodium currents in dorsal unpaired median neurons from the American cockroach *Periplaneta americana*. We show that Ae1a interacts with the domain II voltage sensor and that sensitivity to the toxin is conferred by natural sequence variations in the S1–S2 loop of domain II. The phyletic specificity of Ae1a provides crucial information for development of sodium channel insecticides that target key insect pests without harming beneficial species.

Spider venoms are extremely complex mixtures of proteins, peptides and low molecular weight components such as ions, organic acids, neurotransmitters, polyamines, and nucleotides/nucleosides^1^,^2^,^3^. However, the dominant components of most spider venoms are disulfide-rich peptides, with some venoms containing more than 1000 unique peptides^4^. Most of those peptides exhibit insecticidal activity, consistent with the fact that spider venoms evolved primarily to target insect prey^5^. The principal molecular targets of spider-venom peptides are presynaptic ion channels, with modulation of these channels enabling rapid incapacitation of envenomated prey^3^ and/or deterrence of predators^6^.

Over the past decade there has been a substantial decline in the arsenal of registered chemical insecticides due to the development of resistance in pest insects and de-registration of key insecticides by regulatory authorities due to their perceived threat to the environment and/or human health^3^. This development has stimulated research into eco-friendly alternatives to chemical insecticides, such as insecticidal protein toxins from bacterial entemopathogens^7^,^8^ and insecticidal venom peptides^9^,^10^. The potential advantages of venom peptides over conventional chemical insecticides include their high potency, selectivity for insects, and their presumed low ecological impact. However, the poor oral bioavailability of peptides in comparison to chemical insecticides has long been considered a major disadvantage. Nevertheless, some spider-venom peptides do exhibit oral activity^11^,^12^. Moreover, even for spider-venom peptides that are not orally active, there is still a wide range of possible application strategies that could facilitate their deployment as bioinsecticides^3^,^13^,^14^,^15^.

An ideal bioinsecticide would be specific for the targeted insect pest, without harming beneficial insects. Although most spiders are generalist predators that hunt many types of insect prey, some spider-venom peptides have been isolated that are specific to certain insect orders or even to certain species within an insect order^16^,^17^. For example, we recently demonstrated that the spider-venom peptide β-diguetoxin-Dc1a is lethal to German cockroaches (*Blattella germanica*) but does not induce adverse effects at much higher doses in American cockroaches (*Periplaneta americana*)^18^. Another example of a taxonomically-selective spider-venom peptide is ω-hexatoxin-Hv1a, which is lethal to a wide range of insect pest species^19^,^20^ but inactive against honeybees^17^. A spider-venom peptide related to ω-hexatoxin-Hv1a was recently approved by the United States Environmental Protection Agency for use as a bioinsecticide on a range of crops^14^.

Here we describe the isolation and characterization of a insecticidal peptide (Ae1a) with complex species selectivity from the venom of the African tarantula *Augacephalus ezendami*. Recombinant Ae1a produced by overexpression in the periplasm of *Escherichia coli* was shown to be insecticidal against two dipteran species (*Lucilia cuprina* and *Drosophila melanogaster*) but not the lepidopteran *Helicoverpa armigera*. Electrophysiological studies revealed that the insecticidal activity of the toxin is at least partially due to inhibition of insect voltage-gated sodium (Na_V_) channels. We show that the toxin binds to the voltage sensor in Na_V_ channel domain II and inhibits channel opening by shifting the threshold for channel activation to more positive voltages. We demonstrate that toxin binding is sensitive to residues in the S1–S2 loop of the domain II voltage sensor, and we use this information to make predictions about the species selectivity of the toxin.

## Results

### Isolation of Ae1a

**Figure 1:**
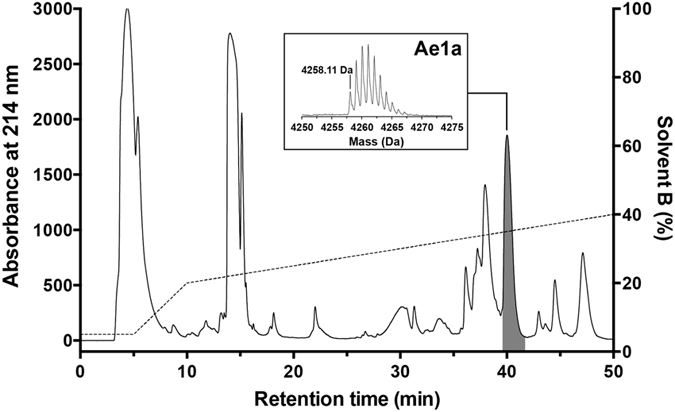
Isolation of Ae1a. Chromatogram resulting from fractionation of crude *Augacephalus ezendami* venom using C_18_ RP-HPLC. The dashed line indicates the gradient of solvent B (90% acetonitrile/0.05% TFA). The shaded peak containing the active peptide Ae1a was lethal when injected into *Drosophila melanogaster*. The inset shows the MALDI MS spectrum of purified Ae1a.

A screen for insecticidal activity by injection into sheep blowflies (*L. cuprina*, *n* = 5) of fractions from reversed-phase (RP) HPLC fractionation of *A. ezendami* venom revealed that the fraction eluting at ~41 min (Fig. 1) rapidly induced paralysis that reversed within 24 h. This fraction contained a single dominant peptide with a monoisotopic mass of 4257.11 Da (Fig. 1, inset). Based on its activity (see below) and source of origin, we named this peptide μ-theraphotoxin-Ae1a (Ae1a) based on the rational nomenclature for spider toxins that is used by UniProt/VenomZone (http://venomzone.expasy.org) and ArachnoServer^21^. N-terminal sequencing of Ae1a returned the sequence GVDKEGCRYLLGACTIDDDCCLHLGCNKKYGHC(G)WD(GWD)T, with the residues in parentheses indicating the most likely assignment for that particular sequencing cycle. A BLAST of this sequence against the ArachnoServer database^20^,^22^ revealed a close match with the sequence of β-theraphotoxin-Cm2a (Cm2a) from the closely related African theraphosid spider *Ceratogyrus marshalli*. Note that *A. ezendami* was only recently reclassified from the genus *Ceratogyrus* into the genus *Augacephalus*^23^. Based on the sequence similarities between Cm2a and Ae1a, glycine is the most likely residue at positions 34 and 37 as predicted from the sequencing data. The resulting sequence, GVDKEGCRYLLGACTIDDDCCLHLGCNKKYGHCGWDGT, yields a calculated monoisotopic oxidized mass of 4111.76 Da, which is 145.35 Da less than the monoisotopic mass measured for the native peptide. We therefore conclude that the C-terminal residue is an amidated phenylalanine, which matches the Cm2a sequence. Thus, the putative sequence for native Ae1a is GVDKEGCRYLLGACTIDDDCCLHLGCNKKYGHCGWDGTF-NH_2_; the calculated monoisotopic mass for this sequence (4257.841 Da) is 0.731 Da higher than the mass measured for native Ae1a. This sequence is 89% identical to Cm2a.

### Production of recombinant Ae1a

**Figure 2:**
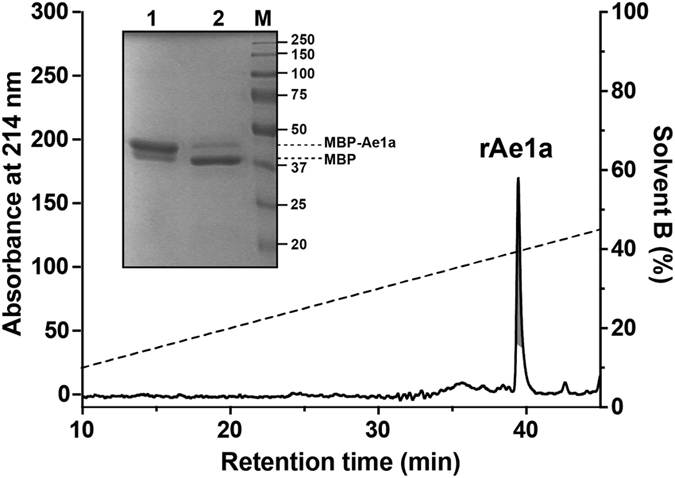
Production of recombinant Ae1a. RP-HPLC chromatogram showing final purification of rAe1a (shaded peak). The dashed line indicates the gradient of solvent B (90% acetonitrile/0.043% TFA). The inset is an SDS-PAGE gel showing cleavage of the MBP-Ae1a fusion protein with TEV protease. Lanes 1 and 2 correspond to pre- and post-cleavage samples, while lane M contains molecular weight markers (masses indicated in kDa). The bands corresponding to uncleaved and cleaved fusion protein are indicated. Note that this image is cropped from the original gel scan, which is shown in its entirety as Supplementary Figure 1, but is otherwise unmodified.

Recombinant Ae1a (rAe1a) was produced via expression of a maltose binding protein (MBP)-Ae1a fusion protein in the periplasm of *E. coli* using a protocol we described previously for production of disulfide-rich venom peptides^24^. The fusion protein was the major soluble protein produced after induction, and cleavage of the fusion protein with tobacco etch virus (TEV) protease yielded free rAe1a with ~75% efficiency (Fig. 2, inset). RP-HPLC purification of the liberated rAe1a resulted in a single major peak with a final yield of ~200 μg rAe1a per liter of bacterial culture (Fig. 2). The recombinant Ae1a peptide was used for all *in vitro* and *in vivo* assays.

### Insecticidal activity of rAe1a

**Figure 3:**
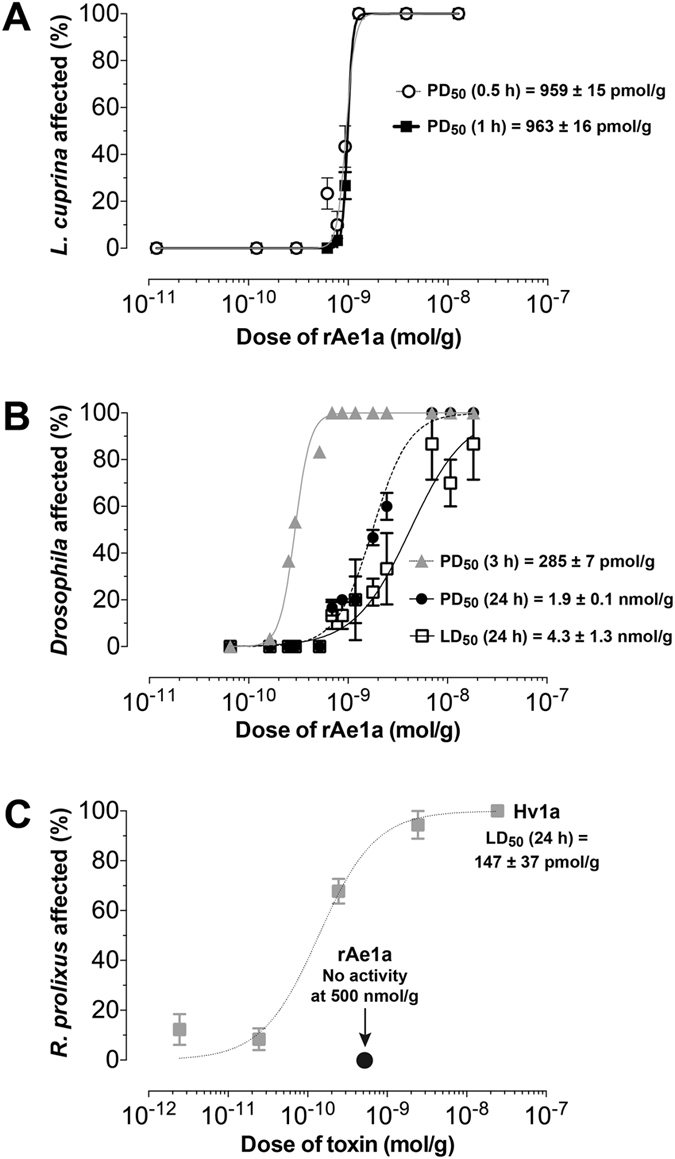
Insecticidal effects of Ae1a. (**A**) rAe1a was injected into blowflies (*Lucilia cuprina*) and paralytic effects were measured 0.5 and 1 h after injection. (**B**) rAe1a was injected into fruit flies (*Drosophila melanogaster*) and paralytic and lethal effects were measured at 3 h and 24 h post injection. (**C**) rAe1a and Hv1a were injected into the triatomine bug *Rhodnius prolixus* and lethality was measured 24 h after injection. PD_50_ and LD_50_ values were calculated as described previously^46^.

Injection of rAe1a into *L. cuprina* induced rapid but reversible paralysis (Fig. 3A). The median paralytic dose (PD_50_; i.e., the dose that paralysed 50% of injected insects) measured at 0.5 and 1 h post-injection was almost identical (959 ± 15 pmol/g and 963 ± 16 pmol/g, respectively), but no paralytic or lethal effects were evident 24 h after injection. In contrast, injection of rAe1a into fruit flies (*D. melanogaster*) induced irreversible paralysis leading to death (Fig. 3B). The PD_50_ measured at 3 h was 285 ± 7.1 pmol/g, which is 3.4-times lower (i.e., more potent) than the PD_50_ in blowflies at 1 h, and at 24 h post-injection the PD_50_ was 1.9 ± 0.1 nmol/g. The median lethal dose (LD_50_; i.e., the dose that killed 50% of injected insects) measured 24 h after injection was 4.3 ± 1.3 nmol/g (Fig. 3B).

Injection of high doses of rAe1a (up to 74.9 nmol/g) into cotton bollworms (*H. armigera* larvae) did not induce paralytic or lethal effects or any significant changes in the weight gain of larvae within the 72 h observation period. Similarly, rAe1a did not elicit any signs of paralysis or lethality in the triatomine bug *Rhodnius prolixus* even at a very high dose of 500 pmol/g (Fig. 3C). In contrast, the well-characterized insecticidal spider-venom peptide ω-HXTX-Hv1a (Hv1a), a selective inhibitor of insect voltage-gated calcium channels^19^,^25^, was potently lethal to *R. prolixus* with an LD_50_ of 147 ± 37 pmol/g (Fig. 3C).

### rAe1a inhibits opening of German cockroach Na_V_ channels

**Figure 4:**
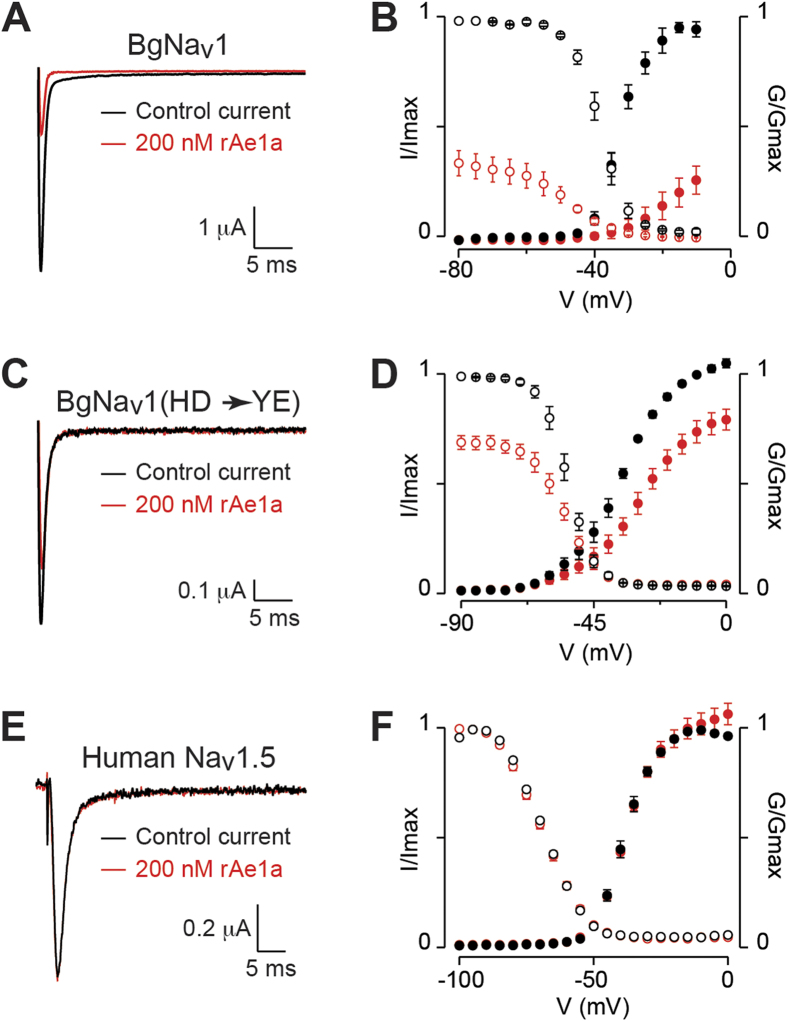
Effect of rAe1a on BgNa_v_1 and hNa_V_1.5. Left panels: Representative traces showing the effect of rAe1a (200 nM) on sodium currents mediated by (**A**) BgNa_V_1, (**C**) mutant BgNa_V_1 (H805Y/D812E), and (**E**) hNa_V_1.5 heterologously expressed in *Xenopus* oocytes. Currents were evoked by a depolarization to −15 mV, with black and red traces corresponding to the current before and after toxin application, respectively. Right panels: Effect of 200 nM rAe1a on normalized conductance-voltage (**G–V**) relationships (G/G_max_) and steady-state inactivation (SSI) relationships (I/I_max_) for (**B**) BgNa_V_1, (**D**) mutant BgNa_V_1, and (F) hNa_V_1.5. G/G_max_ and I/I_max_ are shown by closed and open circles respectively, before (black) and after (red) toxin addition. Normalization was performed relative to the peak current before toxin addition. Oocytes were depolarized by steps of 5 mV from a holding potential of −90 mV up to 5 mV for 50 ms, followed by a depolarizing pulse to −15 mV for 50 ms. Peak current from the initial step series was converted to conductance and normalized to obtain the G-V relationship while peak current from the following −15 mV voltage depolarization step was normalized to yield the SSI relationship. rAe1a inhibited the mutant BgNa_V_1 channel to a lesser extent than wild-type BgNa_V_1, and it had no effect on the human Na_V_1.5 channel. Data points are mean ± SEM, and *n* = 3–5 for all experiments shown.

rAe1a dramatically inhibited currents mediated by the BgNa_V_1 channel from the German cockroach *Blattella germanica* heterologously expressed in *Xenopus* oocytes. At a concentration of 200 nM, rAe1a shifted channel activation to more positive voltages (*V*_1/2_ was shifted from −32.5 ± 0.2 mV to −17.5 ± 0.5 mV; *n* = 4) while steady-state inactivation (or channel availability) was shifted to more negative voltages (*V*_1/2_ was shifted from −38.4 ± 0.1 mV to −47.8 ± 0.3 mV; Fig. 4A,B). These effects are typical of gating modifier sodium channel toxins from spider venoms that interact with the domain II voltage-sensor^26^,^27^,^28^. At mildly depolarizing voltages near the foot of the *G*_Na_–V relationship curve, toxin-induced inhibition was more pronounced when compared to more depolarizing voltages, indicating that BgNa_v_1 can activate with rAe1a bound to one or more voltage-sensor domains (Fig. 4B).

### rAe1a has no effect on American cockroach Na_V_ channels

**Figure 5:**
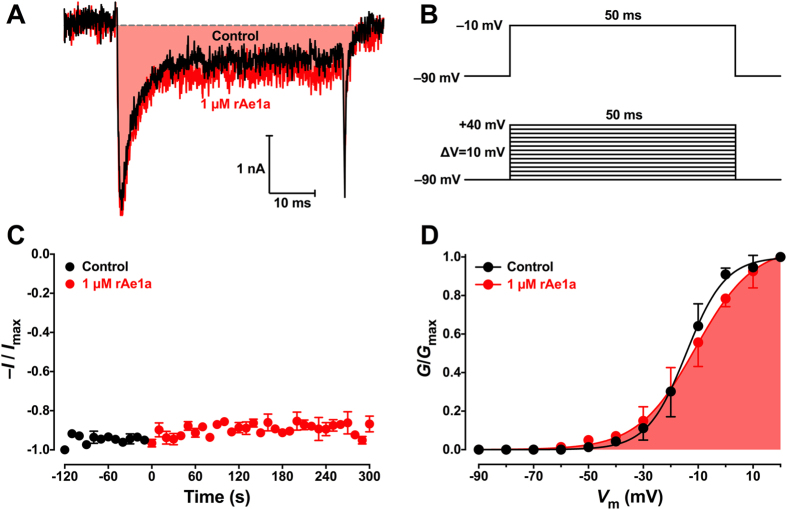
Effect of rAe1a on Na_V_ channel currents in *P. americana* DUM neurons. (**A**) Representative traces showing the effect of 1 μM rAe1a on *I*_Na_ mediated by PaNa_V_1 in *P. americana* DUM neurons. Currents were evoked by depolarizations to −10 mV, from a holding potential of −90 mV (panel **B**), with black and red traces corresponding to the current before, and 5 min after, toxin application, respectively. (**C**) Time course of rAe1a actions on PaNa_V_1. Peak *I*_Na_ were recorded at a rate of 0.1 Hz before (black circles), and for 5 min during, application of 1 μM rAe1a (red circles) and normalized against maximal peak *I*_Na_ (−*1*/*I*_max_; *n* = 3). (**D**) *G*_Na_-*V* relationships in *P. americana* DUM neurons. A voltage protocol with depolarisation steps from −90 mV to +40 mV in 10 mV increments (lower panel **B**) was used to generate families of *I*_Na_. The normalized conductance-voltage (*G*_Na_-*V*) relationship (*G*/*G*_max_) is shown before (black circles) and after (red circles) a 5 min perfusion with 1 μM rAe1a (*n* = 4). Conductance was normalized relative to the peak current before toxin addition.

In striking contrast to its potent inhibition of the cloned BgNa_V_1 channel, rAe1a had no effect on Na_V_ channel currents in dorsal unpaired median (DUM) neurons from *P. americana*. During a standard test pulse to −10 mV from a holding potential of −90 mV (Fig. 5B) we observed only a 8.7 ± 0.4% (*n* = 3, *P* = 0.166, paired Student’s t-test) reduction in peak current amplitude following a 5-min perfusion with 1 μM rAe1a (Fig. 5A,C). To determine whether there were any voltage-dependent alterations in the activation of Na_V_ channel currents, families of *I*_Na_ were elicited using a test pulse that depolarized the cell from *V*_h_ of −90 mV to +40 mV for 50 ms in 10-mV increments (Fig. 5B). Peak *I*_Na_ values were then converted to *G*_Na_ according to Eq. 1 and plotted against membrane potential (*V*) to establish a *G*_Na_-*V* curve. In the absence of toxin, *I*_Na_ activated around −50 mV. This threshold was not significantly altered in the presence of 1 μM rAe1a (Fig. 5D) and the voltage at half maximum Na_V_ channel activation (*V*_1/2_) in control cells was only marginally shifted (+3.1 mV) in the depolarizing direction in the presence of 1 μM rAe1a (control *V*_1/2_ = −15.4 ± 1.7 mV versus toxin *V*_1/2_ = −12.3 ± 2.5 mV, *n* = 4 cells, *p* > 0.5).

### rAe1a has no effect on the human voltage-gated sodium channel Na_V_1.5

Similar to its lack of effect on Na_V_ channel currents in *P. americana* DUM neurons, we found that 200 nM rAe1a did not inhibit currents from, nor affect the gating properties of, the human cardiac voltage-gated sodium channel hNa_V_1.5 expressed in *Xenopus* oocytes (Fig. 4E,F).

### Variations in the DII S1–S2 loop mediate Na_V_ channel sensitivity to Ae1a

**Figure 6:**
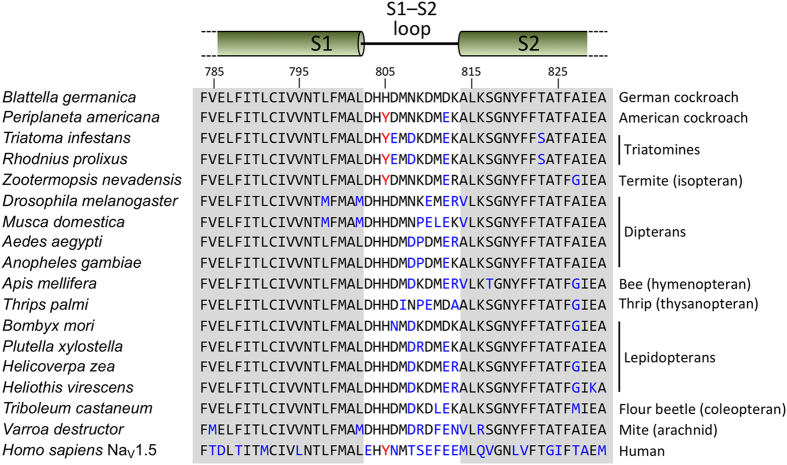
Alignment of the DII S1–S2 region of insect Na_V_ channels and human Na_V_1.5. Regions corresponding to the S1 and S2 transmembrane helices are shaded grey, and the numbering above the sequences corresponds to *Blattella germanica* BgNa_V_1. Sequence changes relative to the BgNa_V_1 sequence shown at the top of the alignment are highlighted in blue. The resistance-conferring Tyr at position 805 is highlighted in red when present. Note the vastly different S1–S2 loop sequence in the human Na_V_1.5 channel (bottom sequence).

Why does Ae1a kill fruit flies but not closely related blowflies, and inhibit sodium channels from German but not American cockroaches? Previous studies have revealed that many gating modifier toxins from spider venoms modulate the activity of Na_V_ channels by interacting with the S3b–S4 paddle motif in the domain II (DII) voltage sensor^18^,^26^,^27^,^28^,^29^,^30^,^31^. However, since this region is identical in the voltage-gated sodium channels from American (PaNa_V_1) and German (BgNa_V_1) cockroaches, variations in this region of the channel cannot explain the complex species selectivity of Ae1a. Thus, we explored whether residues outside this region might contribute towards the lower sensitivity of PaNa_V_1 towards rAe1a. To this end, we expressed in oocytes a previously constructed^18^ mutant of BgNa_V_1 that contains two mutations in the DII S1–S2 loop (His805*Tyr and Asp812*Glu) such that the mutant channel mimics the DII voltage-sensing domain of PaNa_V_1 (see sequence alignment in Fig. 6). We found that this mutant channel is much less sensitive to the effects of rAe1a; in the presence of 200 nM rAe1a, the activation *V*_1/2_ was shifted from −35.5 ± 0.2 mV to −29.3 ± 0.3 mV, while channel availability *V*_1/2_ was only marginally shifted from −53.7 ± 0.1 mV to −54.8 ± 0.1 mV (Fig. 4C,D). These shifts in V_1/2_ are much smaller than those elicited by rAe1a on wild-type BgNa_V_1 (Fig. 4A,B). Thus, these data indicate that natural sequence variations within the DII S1–S2 loop are largely responsible for the susceptibility of insect Na_V_ channels to Ae1a.

## Discussion

Numerous insecticidal toxins have been isolated from the venoms of spiders and scorpions^3^,^9^,^10^,^16^,^32^,^33^ but very few have a high degree of phyletic selectivity. This makes ecological sense as most arachnids are generalist predators that do not specialize on particular prey taxa. However, with respect to their application in agriculture, insecticidal toxins that could specifically target insect pests while sparing beneficial insects would be highly desirable. In this paper we describe a unique spider toxin that has striking taxonomic selectivity.

We used the fruit fly *Drosophila melanogaster* to rapidly screen spider venoms for insecticidal activity. This revealed that the venom of *A. ezendami* is highly toxic to fruit flies, so we decided to focus on this previously unstudied spider venom with a view to discovering novel insecticidal toxins. Microinjection of venoms or toxins into *D. melanogaster* has the advantage that it requires only small volumes (50 nl per fly), which enables the study of scarce venoms from small venomous animals. Given the worldwide availability of *Drosophila* and the general sensitivity of flies towards spider toxins^16^, this assay provides a convenient method for determining the insecticidal potency of venom compounds. However, as discussed below, it should not be used in isolation but rather as part of a taxonomically diverse screening protocol that aims to uncover potentially complex phyletic specificities.

A number of spider toxins have been described that induce irreversible paralysis in sheep blowflies, generally leading to death^34^,^35^,^36^. The insecticidal potency of these toxins varies from an LD_50_ of 198 pmol/g for U_1_-AGTX-Ta1a^36^ to a PD_50_ of 2200 pmol/g for μ-SGTX-Sf1a^34^. All of these previously reported toxins are relatively non-selective, displaying activity against a wide range of insect species. In contrast, we describe here a novel toxin (Ae1a) isolated from the venom of the African theraphosid spider *A. ezendami* that is moderately potent against sheep blowflies (PD_50_ ~ 950 pmol/g) but which has marked species-specific selectivity. The rapid paralysis induced by injection of the toxin into blowflies reverses within 24 h. In striking contrast, Ae1a induces *irreversible* paralysis leading to death in the closely related dipteran species *D. melanogaster*. Moreover, at the molecular level, we found that Ae1a potently inhibits the voltage-gated sodium channel BgNa_V_1 from the German cockroach *B. germanica* but has no significant affect on the orthologous PaNa_V_1 channel from the American cockroach *P. americana*. Thus, Ae1a displays remarkably complex species-specific insecticidal activity.

The selectivity of Ae1a for BgNa_V_1 over PaNa_V_1 channels is reminiscent of the selectivity reported for the insecticidal spider-venom peptide μ-DGTX-Dc1a (Dc1a) isolated from the American desert spider *Diguetia canities*^18^. In order to explore the molecular basis of this species selectivity we examined the ability of Ae1a to inhibit the activity of a BgNa_V_1 channel containing two mutations that were previously reported to determine sensitivity to Dc1a^18^. We found that BgNa_V_1 was much less sensitive to the effects of Ae1a when two mutations (H805Y and D812E) were introduced into the DII S1–S2 loop in order to replicate the S1–S2 loop found in equivalent voltage sensor of PaNa_V_1. Thus, sequence variations within the DII S1–S2 loop at least partly determine the sensitivity of insect Na_V_ channels to effects of Ae1a. Ae1a therefore belongs to a growing class of peptidic Na_V_ channel toxins in which the S1–S2 loop in channel domain II is implicated in toxin binding^18^,^31^. By binding into the cleft between the S1–S2 and S3–S4 loops it is presumed that these toxins impede outward movement of the S4 transmembrane helix that is essential for channel activation^37^.

Figure 6 shows an alignment of the DII S1–S2 region from the Na_V_ channels of a taxonomically diverse group of insects including *D. melanogaster*, which is highly sensitive to Ae1a. Notably, the DII S1–S2 region of DmNa_V_1 contains the same D812E substitution found in *P. americana* and we therefore conclude that the H805Y substitution in PaNa_V_1 is the primary cause of its resistance to Ae1a. Thus, the amino acid at this position in the S1–S2 loop can be used to predict sensitivity to Ae1a. Unfortunately, the sequence of the Na_V_ channel from *H. armigera*, which we found to be resistant to Ae1a, is not yet available. All lepidopteran species with Na_V_ channel sequences available have a histidine at the position equivalent to H805 in *B. germanica* and are therefore predicted to be susceptible to Ae1a; consistent with this prediction, we found that Ae1a was lethal when injected into larvae of the diamondback moth *Plutella xylostella* (data not shown).

Interestingly, based on the alignment shown in Fig. 6, we predict that, aside from cotton bollworms, triatomine bugs (e.g., *Triatoma infestans* and *R. prolixus*) and termites (e.g., *Zootermopsis nevadensis*) are the only insects that are likely to show high levels of resistance to Ae1a. Consistent with this prediction, we found that Ae1a is inactive against the kissing bug *R. prolixus*, an important vector of Chagas disease^38^, whereas this triatomine is highly susceptible to the effects of the spider-venom peptide Hv1a (Fig. 3C).

Ae1a is likely to be a useful bioinsecticide for targeting a wide range of insect pests, although it will be important to assess off-target effects, especially on vertebrates and beneficial insects. In this regard, it is important to note that the DII S1–S2 regions of human and insect Na_V_ channels are quite different, as exemplified by hNa_V_1.5 in Fig. 6. Moreover, five of the nine human subtypes (hNa_V_1.1–hNa_V_1.5) contain the resistance-conferring H805Y substitution found in PaNa_V_1. Thus, we predict that humans will be resistant to the effects of Ae1a. In line with this prediction, we found that Ae1a is completely inactive against the human Na_V_1.5 channel (Fig. 4E,F).

Commercial insecticides such as pyrethroids, indoxacarb and DDT target insect Na_V_ channels and numerous insect species have developed target-site resistance to these agrochemicals (so-called *kdr* and super-*kdr* mutations)^39^. It therefore might be argued that Na_V_ channels are not an ideal molecular target for new insecticides. However, whereas these chemical insecticides target the pore region of insect Na_V_ channels and prevent channel closure, we have shown that Ae1a targets the DII voltage sensor and inhibits the channel via a distinctly different mechanism of action. Moreover, it has been demonstrated that a pyrethroid–resistant strain of the tobacco budworm *Heliothis virescens* is actually more vulnerable than susceptible strains to the effects of a recombinant baculovirus expressing a gating modifier scorpion toxin (AaIT) that also binds to the DII voltage sensor of insect Na_V_ channels^40^. Thus, *kdr* mutations are not expected to impact susceptibility to Ae1a.

In conclusion, we isolated and characterized a novel insecticidal toxin (Ae1a) from the venom of the theraphosid spider *A. ezendami*, which is endemic to Mozambique. The insecticidal activity of Ae1a results from inhibition of insect Na_V_ channels. Mapping of the toxin binding site to specific residues within the domain II voltage sensor of insect Na_V_ channels enabled prediction of which species are likely to be susceptible or resistant to Ae1a. In combination with recent seminal advances in understanding the structure of Na_V_ channels^41^,^42^,^43^, this work provides a solid foundation for the rational engineering of insecticidal peptides with well-defined species selectivity.

## Methods

### Venom collection

Venom was extracted from female *A. ezendami* held in a private collection in Germany via mild electrical stimulation of the chelicerae^44^. Venom was lyophilized and stored at −20 °C until further use.

### High performance liquid chromatography

Lyophilized *A. ezendami* venom (1 mg) was fractionated on a Phenomenex Jupiter C_18_ reverse-phase (RP) analytical HPLC column (250 × 4 mm, 5 μm; Phenomenex, Sydney, NSW, Australia) connected to a Shimadzu Prominence HPLC system using the following gradient of solvent B (90% acetonitrile/0.05% trifluoracetic acid (TFA)) in solvent A (0.05% TFA in H_2_O): 5% B for 5 min, 5–20% B over 5 min, 20–40% B over 40 min. The flow rate was 1 ml/min.

### Mass spectrometry

The mass of native and recombinant toxins was determined using matrix-assisted laser desorption/ionization time-of-flight mass spectrometry (MALDI-TOF MS) using a Model 4700 Proteomics Analyser (Applied Biosystems, Foster City, CA, USA). Toxin samples were mixed 1:1 (v:v) with α-cyano-4-hydroxy-cinnamic acid matrix (6 mg/ml in 50/50 acetonitrile/H_2_O with 5% formic acid) and MALDI-TOF spectra were acquired in positive reflector mode. All reported masses are for monoisotopic [M + H]^+^ ions.

### N-terminal sequencing

Ae1a was solubilized in 25 mM ammonium bicarbonate, reduced using dithiothreitol (25 mM) at 56 °C for 30 min, then alkylated using iodoacetamide (55 mM) at room temperature for 30 min. Fully reduced and alkylated Ae1a was then purified via RP-HPLC using a Zorbax 300SB-C18 column (3 × 150 mm). The purified reduced/alkylated Ae1a was then loaded onto a precycled Biobrene disc and N-terminal sequencing via Edman degradation was performed by the Australian Proteome Analysis Facility (Sydney, NSW, Australia) using an Applied Biosystems 494 Procise Protein Sequencer.

### Production of recombinant Ae1a

Recombinant Ae1a was produced by expression in the periplasm of *Escherichia coli* using a previously described protocol^24^. In brief, a synthetic gene encoding Ae1a, with codons optimized for expression in *E. coli*, was produced and cloned into a variant of the pLic-MBP expression vector by GeneArt (Invitrogen, Regensburg, Germany). This vector encodes a MalE signal sequence for periplasmic export, a His_6_ tag for affinity purification, a maltose-binding protein (MBP) fusion tag to aid solubility, and a tobacco etch virus (TEV) protease recognition site directly preceding Ae1a. The plasmid encoding Ae1a was transformed into *E. coli* strain BL21(λDE3) for recombinant toxin production. Protein expression and purification were performed as described^24^ with minor modifications. In summary, a 50-mL starter culture grown overnight in Luria-Bertani broth at 37 °C with shaking (~200 rpm) was used to spike a 2 L culture the following day. After the culture reached an OD_600_ of ~1.0, toxin gene expression was induced via addition of 500 μM IPTG. Cells were grown at 37 °C for a further 3 h before harvesting by centrifugation for 15 min at 10,500 *g*. The His_6_-MBP-toxin fusion protein was extracted from the periplasm by cell disruption at 27 kPa (TS Series Cell Disrupter, ConstantSystems Ltd, Daventry, UK), then captured by passing the extract (buffered in TN buffer: 40 mM Tris, 450 mM NaCl, pH 8.0) over Ni-NTA Superflow resin (Qiagen Pty Ltd, Chadstone, VIC, Australia). Proteins bound non-specifically were removed by washing twice with TN buffer containing 15 mM imidazole, then the fusion protein was eluted with TN buffer containing 300 mM imidazole. The eluted fusion protein was concentrated to 5 ml using a 30 kDa cut-off centrifugal filter, then the buffer was exchanged to TN to remove imidazole. The fusion protein solution was diluted to 10 mL with TN then the fusion protein was cleaved overnight at room temperature following addition of ~100 μg TEV protease in the presence of reduced and oxidized glutathione (0.6 mM and 0.4 mM, respectively) to maintain protease activity^24^. The excised His_6_-MBP and TEV protease were precipitated by addition of 0.1% TFA, then the sample was centrifuged at 14,100 *g*. The supernatant was filtered using a 0.45 μm syringe filter (EMD Millipore, Billerica, MA, USA), then recombinant Ae1a (rAe1a) was further purified using RP-HPLC on a Phenomenex Jupiter C_4_ semi-preparative column (250 × 10 mm, 10 μm) connected to a Prominence HPLC system (Shimadzu Scientific Instruments, Rydalmere, NSW, Australia) using the following gradient of solvent B (90% acetonitrile/0.043% TFA) in solvent A (0.05% TFA in H_2_O): 5% B for 5 min, 5–45% B over 40 min. The flow rate was 5 ml/min.

### Insecticidal activity of Ae1a

#### Sheep blowflies

rAe1a was dissolved in insect saline^45^ and injected into the ventro-lateral thoracic region of adult sheep blowflies (*L. cuprina*; mass 26.8–29.7 mg) as described previously^35^. A maximum of 2 μl was injected per fly using a 1.0 ml Terumo Insulin syringe with 29-gauge needle fitted to an Arnold hand micro-applicator (Burkard Manufacturing Co. Ltd., Rickmansworth, England). After injection, flies were individually housed in 2-ml tubes and paralytic effects determined after 0.5, 1 and 24 h. A total of three assays were performed, and for each assay nine doses of rAe1a (*n* = 10 flies per dose) and the appropriate control (insect saline; *n* = 20 flies each) were used. PD_50_ values were calculated as described^46^.

#### Fruit flies

The effect on rAe1a on fruit flies was determined using a previously described method^47^,^48^. rAe1a was dissolved in 100 mM ammonium acetate buffer (pH 6.1) and injected into the lateral thoracic region of adult female *D. melanogaster* using a glass capillary connected with silicone tubing to a 10 ml plastic syringe. The injection volume per fruit fly was calibrated to be 50 nl for each glass capillary. After injection, fruit flies were housed in 68-ml plastic tubes (containing a cotton piece soaked in 0.5% sucrose solution) in groups of 10 (all receiving the same treatment) and monitored for signs of paralysis or lethality at 3 and 24 h post-injection.

#### Cotton bollworms

We examined the effect of rAe1a on cotton bollworms (i.e., larvae of *H. armigera*) supplied by AgBiTech (Glenvale, QLD, Australia). Larvae received an injection into the lateral thoracic region and were observed for paralytic or lethal effects at 0.5, 1, 3, 24, 48, and 72 h after injection. Larvae were kept on artificial diet (AgBiTech) in standard 6-well plates and their mass was measured 24, 48 and 72 h after the injection.

#### Triatomine bugs

Toxicity against *R. prolixus* was measured using the method described by Luna *et al*.^49^ with some modifications. Microinjection needles, made by pulling borosilicate glass capillaries with a needle puller (Narishige, PC-10, Japan), were fitted onto a Nanoject II auto injector (Drummond Scientific, Broomall, PA, USA). Ae1a, Hv1a, or PBS (*n* = 7–10 per dose, 138 nl volume) were injected into the thorax of second instar *R. prolixus* nymphs (average mass 1.4 mg) via articulation of prothoracic coxa. Synthetic Hv1a was kindly supplied by Vestaron Corporation (Kalamazoo, MI, USA). Three separate sets of experiments were performed for each compound. After injection, bugs were transferred to plastic cages and stored at 26 °C with 50% relative humidity. All bugs were checked at 24 h post-injection for signs of paralysis or lethality.

#### Electrophysiology using insect neurons

Dorsal unpaired median (DUM) neurons were isolated from unsexed adult American cockroaches (*P. americana*) as described^35^. Briefly, terminal abdominal ganglia were removed and placed in normal insect saline (NIS) containing 180 mM NaCl, 3.1 mM KCl, 10 mM *N*-hydroxyethylpiperazine-*N*-ethanesulfonic acid (HEPES), and 20 mM D-glucose. Ganglia were then incubated in 1 mg/ml collagenase (type IA) for 40 min at 29 °C. Following enzymatic treatment, ganglia were washed twice in NIS then resuspended in NIS supplemented with 4 mM MgCl_2_, 5 mM CaCl_2_, 5% foetal bovine serum and 1% penicillin/streptomycin (Life Technologies, VIC, Australia) (NIS+) and triturated through a fire-polished Pasteur pipette. The resultant cell suspension was then distributed onto 12-mm diameter glass coverslips pre-coated with 2 mg/ml concanavalin A (type IV). DUM neurons were maintained in NIS+ at 29 °C and 100% humidity.

Ionic currents were recorded from DUM neurons in voltage-clamp mode using the whole-cell patch-clamp technique employing version 10.2 of the pCLAMP data acquisition system (Molecular Devices, Sunnyvale, CA, USA). Data were filtered at 10 kHz with a low-pass Bessel filter with leakage and capacitive currents subtracted using *P*–*P*/4 procedures. Digital sampling rates were set between 15 and 25 kHz depending on the length of the protocol. Single-use 0.8–1.5 MΩ electrodes were pulled from borosilicate glass and fire-polished prior to current recordings. Liquid junction potentials were calculated using JPCALC, and all data were compensated for these values. Cells were bathed in external solution through a continuous pressurized perfusion system at 1 ml/min, while toxin solutions were introduced via a wide-bore gravity-fed perfusion needle at ~80 μl/min (Automate Scientific, San Francisco, CA, USA). Control data were not acquired until at least 10 min after whole-cell configuration was achieved to eliminate the influence of fast time-dependent shifts in steady-state inactivation resulting in run-down of sodium currents (*I*_Na_) from *P. americana* Na_V_ (PaNa_V_1) channels. All experiments were performed at ambient temperature (20–23 °C). To record *I*_Na_, the external bath solution contained (in mM): NaCl 80, CsCl 5, CaCl_2_ 1.8, tetraethylammonium chloride 50, 4-aminopyridine 5, HEPES 10, NiCl_2_ 0.1, and CdCl_2_ 1, adjusted to pH 7.4 with 1 M NaOH. The pipette solution contained (in mM): NaCl 34, CsF 135, MgCl_2_ 1, HEPES 10, ethylene glycol-bis(2-aminoethylether)-*N*,*N*,*N*′,*N*′-tetraacetic acid (EGTA) 5, and ATP-Na_2_ 3, adjusted to pH 7.4 with 1 M CsOH. To eliminate any influence of differences in osmotic pressure, all internal and external solutions were adjusted to 400 ± 5 mOsmol/l with sucrose. Experiments were rejected if leak currents exceeded 1 nA or currents showed signs of poor space clamping. Peak current amplitude was analysed offline using AxoGraph X v1.5.3 (Molecular Devices). All curve-fitting was performed using Prism 6 (GraphPad Software Inc., CA, USA). Nonlinear regression and a least-squares method was used for fitting *G*_Na_/V curves. Sample means were compared using a paired Student’s *t-*test. A test was considered to be significant when *p* < 0.05. All data are mean ± SEM of *n* independent experiments. Values for sodium conductance (*G*_Na_) were calculated according to the following equation:
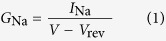
where *I*_Na_ is the absolute value of the sodium current at a given test potential (*V*) and *V*_rev_ is the reversal potential. The values of *G*_Na_ and *V* were then fitted to a Boltzmann equation :
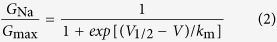
where *G*_max_ is maximal *G*_Na_, *V*_1/2_ is the half- maximal conductance, and *k*_m_ is the slope factor.

### Electrophysiology using cloned Na_V_1 channels

cRNA encoding BgNa_V_1 from *Blattella germanica*^50^ and hNa_V_1.5 (Origene, Rockville, MD, USA) was synthesized using T7 polymerase (mMessage mMachine kit, Life Technologies, USA) after linearizing the fully-sequenced DNA. BgNa_V_1 and hNa_V_1.5 were expressed in *Xenopus* oocytes together with either the TipE subunit^51^ or human β1 subunit (1:5 molar ratio), respectively. Oocytes were incubated at 17 °C in 96 mM NaCl, 2 mM KCl, 5 mM HEPES, 1 mM MgCl_2_ and 1.8 mM CaCl_2_, 50 μg/ml gentamycin, pH 7.6 with NaOH, and currents were measured 1–2 days after cRNA injection using the two-electrode voltage-clamp recording technique (OC-725C, Warner Instruments, Hamden, CT, USA) with a 150-μl recording chamber. Data were filtered at 4 kHz and digitized at 20 kHz using pClamp 10 (Molecular Devices). Microelectrode resistances were 0.5–1.5 MΩ when filled with 3 M KCl. The external recording solution contained 100 mM NaCl, 5 mM HEPES, 1 mM MgCl_2_ and 1.8 mM CaCl_2_, pH adjusted to 7.6 with NaOH. All experiments were performed at room temperature (~22 °C). Leak and background conductances, identified by blocking the channels with tetrodotoxin, were subtracted for all currents shown. Voltage–activation relationships were obtained by measuring steady-state currents and calculating conductance as described above. Protocols for other measurements are described in figure legends. After addition of rAe1a to the recording chamber, equilibration between toxin and channel was monitored using weak depolarizations elicited at 5-s intervals. Off-line data analysis was performed using Clampfit 10 (Molecular Devices) and Origin 8.0 (OriginLab, Northampton, MA, USA).

## Additional Information

**How to cite this article**: Herzig, V. *et al*. Molecular basis of the remarkable species selectivity of an insecticidal sodium channel toxin with from the African spider *Augacephalus ezendami*. *Sci. Rep.* **6**, 29538; doi: 10.1038/srep29538 (2016).

## Supplementary Material

Supplementary Information
