# Supplementary material for: Molecular basis of the remarkable species selectivity of an insecticidal sodium channel toxin from the African spider Augacephalus ezendami
Source: Sci Rep. 2016 Jul 7;6:29538. doi: 10.1038/srep29538 (PMC4935840; doi:10.1038/srep29538)
Supplement: Supplementary Information [file srep29538-s1.pdf]

Supplementary Data

Molecular basis of the remarkable species selectivity of an insecticidal sodium channel toxin from the African spider *Augacephalus ezendami*

Volker Herzig, Maria Ikonomopoulou, Jennifer J. Smith, Sławomir Dziemborowicz, John Gilchrist, Lucia Kuhn-Nentwig, Fernanda Oliveira Rezende, Luciano Andrade Moreira, Graham M. Nicholson, Frank Bosmans, and Glenn F. King

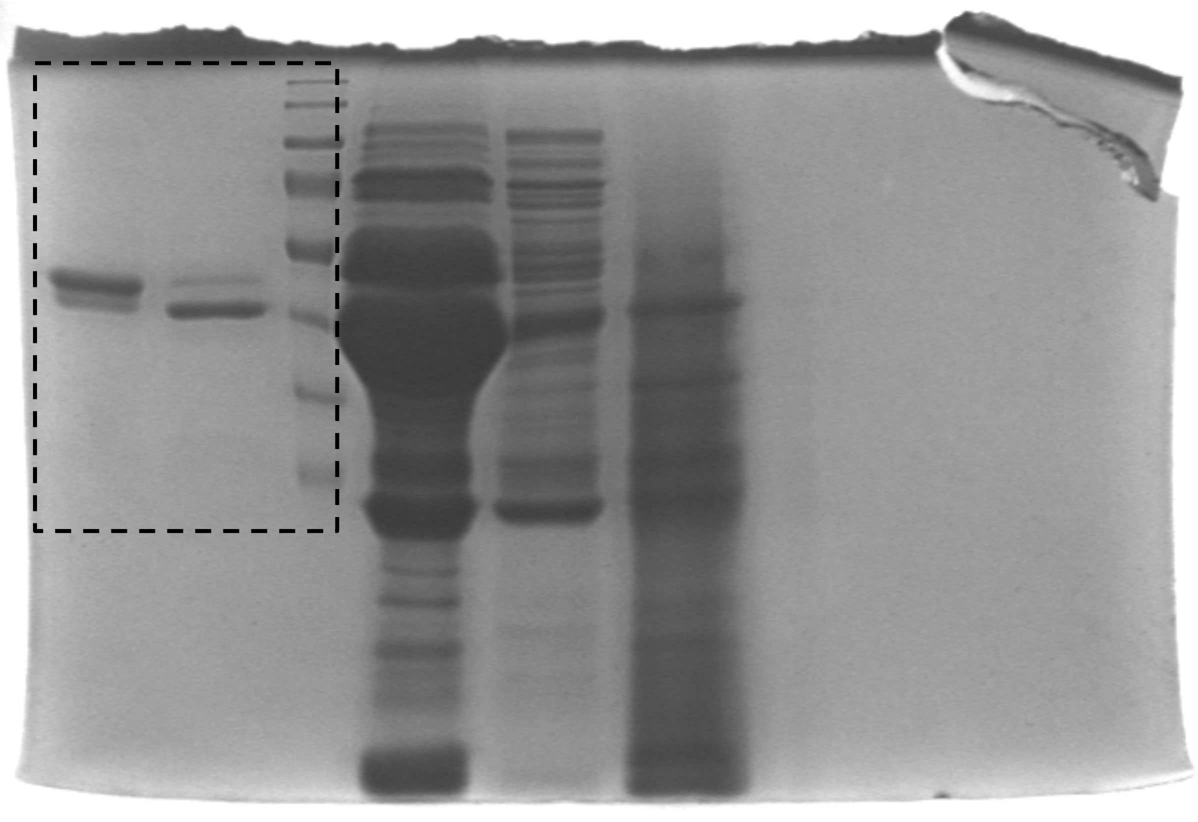

Supplementary Figure 1: Complete scan of SDS-PAGE gel used to generate the cropped image shown as an inset in Figure 2. The cropped region is indicated by the dashed lines. The other gel lanes are not relevant to the current study.
